# Editorial: T regulatory cells: mechanisms and therapeutical advances

**DOI:** 10.3389/fimmu.2026.1947694

**Published:** 2026-08-07

**Authors:** Gloria Soldevila, Maria-Luisa Alegre, Karina Pino-Lagos

**Affiliations:** 1 Department of Immunology, Biomedical Research Institute, National Autonomous University of Mexico, Mexico City, Mexico; 2 National Laboratory of Flow Cytometry, Biomedical Research Institute, National Autonomous University of Mexico, Mexico City, Mexico; 3 Department of Medicine, University of Chicago, Chicago, IL, United States; 4 Facultad de Medicina, Centro de Investigación e Innovación Biomédica CiiB), Universidad de los Andes, Santiago, Chile

**Keywords:** autoimmunity, cell therapy, extracellular vesicles, GvHD, leukemia, metabolic reprogramming, miRNA, neurodegenerative diseases

The assembly of this Research Topic was prompted both by the awarding of the 2025 Nobel Prize to work on FoxP3^+^ regulatory T cells (Tregs) and by the increasing attention these cells have received in a variety of pathological conditions. In parallel with technological advances, FoxP3^+^ Treg cellular therapies are under investigation to treat different diseases.

Here, we introduce a group of manuscripts that include a Hypothesis & Theory article by Bai et al. exposing evidence to support the main idea that Tregs’ modes of suppression are dependent on TCR signaling strength (discussing antigen specificity and bystander suppression), as well as nine reviews and seven original manuscripts. Review articles discussed a wide range of topics from Treg biology (reviewing miRNAs^2^ and metabolic reprogramming^3^) to Tregs in the context of pathology (autoimmunity^4^, hypoxia^5^, leukemia^6^, wound healing^7^, tissue repair and regeneration^8^ and tumor immunity^9^) to Tregs’ clinical applications^10^.

Interestingly, most of articles under Original Research classification cover investigation on human Tregs. In this case, Nirmala et al. reported a comprehensive phenotypic characterization (31 markers by flow cytometry) of human Tregs obtained from different sites (cord-blood, peripheral blood, and thymus) concluding that cord-blood contains the most homogenous Treg pool. Regarding Tregs in the context of disease, Kossack et al. reported an immune monitoring study of COVID-19 patients, which found a correlation between COVID-19 severity, Treg phenotypes, and suppression activity, where severe patients contained Tregs with disrupted function. Wang et al^13^. investigated Tregs from patients with Sjögren’s disease, revealing that Treg numbers are decreased whereas expression of cell death-related molecules and apoptosis are increased in these cells. Continuing with the effort to achieve a good Treg product (suppressive cells with stable function), Alvarez-Salazar et al. and Requejo Cier et al. demonstrated that Tregs exposed to Vitamin C or altered with CRISPR-Cas9 technology to avoid susceptibility to tacrolimus maintained an “untouched” Treg signature, opening new possibilities for their application in the transplantation field.

From an experimental standpoint, Moya-Guzmán et al. demonstrated that different Treg subsets (namely, thymic Tregs [tTregs], induced Tregs [iTregs] and Retinoic acid-iTregs [RA-Tregs]) all produced small extracellular vesicles (sEVs) with *in vitro* suppressive activity. This suppression was at least dependent on death of target cells, with sEVs from tTregs and from RA-Tregs being the most cytotoxic. Last, Beguin et al. described a new protocol to test human Tregs *in vivo* using a xeno- and allogeneic graft-versus-host disease (GvHD) model, as many human Tregs’ function and permanence can be lost or compromised *in vivo*. The study shows that infused human Tregs engraft in the animals and display suppressive function that can be tracked over time.

Together, these contributions capture the dual character of the current Treg field: a maturing understanding of basic biology from TCR signaling and metabolic reprogramming to the mechanisms of suppression, alongside a concerted push toward the clinic. The emphasis on human Tregs across most of the original research, the attention to product stability under pharmacological and genetic manipulation, and the development of improved *in vivo* testing models all reflect a shared priority: translating decades of foundational work into reliable therapies. As the 2025 Nobel Prize underscores, the questions first raised about how the immune system tolerates self-remain timely and exciting. We hope this Research Topic helps orient readers to both the open biological questions and the practical challenges that will shape the next phase of Treg research.

